# Lyssa and the Pasteur Fiasco†Read before the Society of Medical Jurisprudence and State Medicine, October 13th, 1887.

**Published:** 1888-01

**Authors:** N. E. Brill

**Affiliations:** New York


### Art. VI.—LYSSA AND THE PASTEUR FIASCO.†

BY N. E. BRILL, A. M., M. D.
*New York.*

Even in the developmental history of disease we see, curiously enough, the reflection of the struggle for existence in the human species. The most important factors in the human mental organization, which tend to keep alive those best fitted to cope with the obstacles which meet them in their struggle for life, are the instincts and their

† Read before the Society of Medical Jurisprudence and State Medicine, October 13th, 1887.

derivations, the emotions. Fear, dread and awe have had their influence in the production of a condition-complex, which has resulted in the formation of an alleged infectious disease –hydrophobia, or, better, lyssa—and which is but a transformation of what was originally a single emotion. It is to this emotion, or to the instinct which produced it, that we must ascribe all those phenomena, which, culminating in the disease mentioned, have caused the obliteration from this mundane sphere of those who were least able to cope with the difficulties of their surroundings.

You may ask : How can this be ? It requires but a little search into historical records to convince you of the fact that lyssa is but a reproduction of the same belief, perhaps, in a form modified by the progress of thought which evolution has brought to a more material basis—a belief which culminated in the sixteenth and seventeenth centuries in what has been erroneously termed lycanthropy. At that time it was supposed that the devil, to further his own ends, had the power of transforming the individual, man, into the form of some lower animal, usually the wolf, hence the term of lycanthropy, of which the modern English, "turn-coat," is another name, but which has long been divested from its original meaning. This metamorphosis was considered to be a voluntary one. Richard Verstegan wrote* "the were-wolves are certayne sorcerers who, having anoynted their bodies with an oyntment which they make by the instinct of the devill, and putting on a certayne inchaunted girdle, doe not onely unto the view of others seeme as wolves, so long as they weare the said girdle. And they doe dispose themselves as very wolves, in wourrying and killing most of humane creatures." Upon the interesting psychological aspect of this subject I shall not touch, excepting in so far as to support the view that was laid down in the beginning of this paper, and will reserve it, perhaps, for some future occasion.

You will all smile at this belief which you in this country call a delusion, but you still adhere firmly to your convic-

---

* Restitution of Decayed Intelligence, 1628, quoted in Reynold's System of Medicine.

tion in the existence of what is none else than the same delusion : the attribution of the characteristics of the dog to one suffering from lyssa.

The progress of thought is not able to kill universally instinctive dread. When one is exposed to the view of some phenomenon which fills him with awe or fear, if he have a mental development of sufficient stamina, he will seek to discover the cause and, failing, will ascribe it to the unknowable ; but if he be ignorant the emotion of dread will overcome his deficiently developed mental faculties, and then the devil comes into play. So it was and so it remains. The untutored mind of the Middle Ages could not explain the phenomena of insanity and ascribed to the influence of the devil the condition of the poor unfortunates afflicted with that disease.

Hysteria existed in those days as well as in the present, and what would be more natural for those afflicted with that trouble to adopt the belief of their time ; and while suffering from these attacks, be were-wolves or dogs, or any other animal they chose, biting, stamping, chafing and churning their saliva into froth ? Or, again, what would be more natural than for one who was bitten by a wolf or dog to imagine that the animal biting him was a lycanthrope possessed of the evil spirit who inoculated him with the same characteristics, which should doom the involuntary sufferer to everlasting punishment and his soul to hell fire ? This is not a fanciful speculation of mine, but a thought which has many elements of truth and fact to support it, and one to which we must turn to find the origin of the disease which has engaged the attention of physicians so earnestly during the past two years. But you may say that lyssa was known from the time of Homer. Admitted, yet the belief in lycanthropy or in the metamorphosis of man and the gods into other animals is just as old and existed in some form in the most ancient races. Was not Lycaon changed by Zeus into a wolf ? Did not Jupiter Ammon appear as a ram in the deserts of Libya ? Was not Romulus suckled by a wolf ? And so on in the traditional history of every race some such form of belief existed.

That the basis for the foundation of lyssa and its presence in our nosology is flimsily constructed and established by the weak mental organization of individuals who were controlled by a superstition begotten of fear, is well supported by the fact that the alleged disease had and has even in the present day no well defined criteria, no symptomatology which can be stamped as individual. Its alleged characteristics alone have remained, namely, certain mental changes and the transformation of the character of the individual into that of a rabid animal. Even the last symptom is not constant.

True disease, in other words the collection of symptoms and signs which a morbid change in some element of our organization produces, never varies. The history and course of diphtheria ages ago was the history and course of diphtheria to-day. Small pox was the same in the seventeenth century as it is to-day. And why ? For the simple reason that their criteria are dependent upon recognized stable morbid processes. But if we compare the clinical history of hydrophobia or lyssa of the first medical writers or of even those of a few years ago with that of the present, we will be struck by the integral changes which have occurred in its symptomatology, and must recognize that a constantly changing set of symptoms and signs cannot be due to any fixed morbid agency. What is then the inference to be drawn ? Simply that there is no sound organic foundation upon which to rest its place in the classification of diseases due to a morbid agency. Later I shall attempt to offer an explanation of the phenomena presented by this affection.

The first authentic recognition of the disease was by Democritus, who is thus described by Celius Aurelianus*: *Etenim Democritus qui Hippocrati convixit, non solum hanc memoravit passionem, sed etiam ejus causam tradidit cum de opisthotonicis scriberet.* It will be thus seen that Democritus considered the *tetanic* condition of opisthotonos as the chief characteristic of the disease. With

* C. Aurelianus, De morbis acutis et chronicis, lib. III., Cap. IX. XVI.

the exception of a case to be hereafter mentioned, I can find no other writer who has mentioned this condition as one of the signs of rabies. In fact Bollinger* denies that trismus ever occurs or that a general tonic spasm such as is characteristic of tetanus is ever present. Indeed Fagge† asserts that he never met with any recorded case in which complete opisthotonos was present. J. Law‡ makes no mention of any tetanic condition, and asserts that the clonic spasm of the pharyngeal and laryngeal muscles is accompanied by a sound similar to the bark of the dog. Fagge says that the barking noise made by sufferers from hydrophobia appears to be fabulous. Some authors‖ claim that deglutition is impossible not only during the spasms but in the intervals, others enumerate and record cases in which fluids have not only been swallowed during the intervals but at the very height of the convulsive seizure.

But to show the great contradictions in the clinical his tory of the disease, let me give briefly a few cases as occurred forty years ago and a few of the present day:

Case 1¶. Patient Forget. His disease began by *agitation of the right hand,*** December 13th, 1841. This agitation was most marked in the two first fingers. *Transitory contortion of the whole body* followed. He told his comrade that he was not master of his movements and that he had the incontrollable impulse to use his hand against somebody. *He took all sorts of drink, consuming water, beer and absinthe.*

December 15. Profuse perspiration. Eyes animated, face pale, pulse intermittent, with tumultuous heart action; complains of the sensation of a heavy bar across his chest, yet *respiration is free*; lips red and covered by a thick saliva, which he is constantly rubbing away or spitting about; ardent thirst; *reason clear*; agitation when water was given; cried that he was lost, that he had to die; tries to bite, continually saying he will not bite. After the fit, is sad, affectionate, asks pardon of those about him, and begs to be cured. The physician bled him but could not finish the operation on account of the patient's extreme agitation. There was total dysphagia. After a horrible night, the patient died at Charenton.

---

* Ziemssen's Handbuch der spec. Pathol. und Therapie, Bd. III., pp. 563-573, 1874.

† C. Hilton Fagge—Practice of Medicine, 1886, p. 676.

‡ James Law, F. R. C. V. S. Pepper's System of Medicine. 1885, p. 886 *et seq.*

‖ John Gamgee and Arthur Gamgee. Reynold's System of Medicine. Eng. Ed., 1876, p. 341.

¶ Annales de Medico Psychologiques, 1843, Vol. II., p. 132.

** Italics mine.

The autopsy, according to the physician's statement, presented negative results, the doctor " never having seen healthier organs."

Of this patient it is said that he does not remember having been bitten, but his wife asserts that he had been bitten in the middle finger of the right hand the year before by a dog whom he had found and taken home, and who refused food, lay in a corner and died two days later.

Case 2*. A young man introduced his hand into a dog's mouth to make him eat some meal.

A month after the patient was seized with violent pains in the hand followed by the development of rabies and death.

Autopsy : Recent and old cicatrices of the hand.†

The wonderful conclusion of the physician as the result of this finding being "that this young man must have contracted rabies without having been bitten and where there was no moral impression present."

In a case‡ of Dr. Garan presented before the Societé Royale and which was discussed thoroughly the following conclusion by M. Dupuy was reached : "Hydrophobia is not a constant symptom of rabies. The *Sublingual vesicles* observed by Sr. Marochetti have not been seen by any other observer." The Societé Royale de Medicine rejected this latter conclusion, yet M. Dupuy has been confirmed by all subsequent scientists.

Case 3.‖ Period of incubation eleven months. *Prodroma are specially mentioned as being like those of any other acute disease.* The patient, a child of seven years, was previously gay, laughing and playing with its brothers. Sudden onset, tongue dry, yellow coating ; *intense pain in right iliac fossa* ; some heat of skin ; acceleration of pulse ; *continued dysuria.*

Next day, 8 A. M., the abdominal pain and dysuria had disappeared. It took no fluid during the day and obstinately refused to take a single drop of water during the night. It could not swallow and *when its parents insisted* that it should take its *medicine, it fell into convulsions* after a futile attempt to obey. *A regular opisthotonos* occurred in bringing water towards the child. (These not spontaneous, but brought on by sight of water) ,¶ they cease when the water is removed. The child succeeded in taking twice of its medicinal drink without convulsions, warm baths having been given before. After these symptoms there came a terrible nervous spasm and later the old cicatrix began

---

* Annales de Medico-Psychologiques, 1843, Vol. II., p. 468.

† The reporter, Dr. Segalas, evidently arguing his case, says that the young man was unaware that the dog was mad, that the man had no fear on the subject, not even having been aware that he had been bitten, and says that there was no mark of a bite noticed by the patient.

‡ Ann. de Med. Psychologiques, p. 303.

‖ Opus cit., p. 492.

¶ Parenthesis the writer's.

swell up. Viscid saliva about lips, delirium, child chasing dust on its bed; *took ice cream* with delight, later this also was rejected; tore, bit, raved. The bitten leg was paralysed, cicatrix, and its neighboring parts swelled, and the child died in coma.

There was a hemorrhage from the bowels when the pain in the iliac fossa was relieved. The mother said they had not insisted upon killing the dog because there was not the slightest trouble with it, and it afterwards ran away.

Not one of these cases deserves to be recognized as an example of lyssa, although the most of the clinical features are identical to those usually attributed to that disease. The first case was clearly a psychosis and the patient was in the best place for him, namely, in the insane asylum of Charenton.*

In the second case, how there could be a recent and old cicatrix on the hand after a month is a problem which no one but the reporter, Dr. Segalas, alone can solve. His conclusion is worthy of his description of the disease.

Dr. Aubanel's case is clearly not one of lyssa, but seems to be a pure one of typhoid fever.

Of the recent cases the following are interesting:

Case 4.† Dr. Ullerholzner reports a child of eleven years and ten months old who was bitten on the lips by a dog. The wound was cauterized in less than one-half hour after the injury, and healed in fourteen days. After an incubation (?) of twenty-four days, the disease began with chilly sensations, depression, thirst, hydrophobia, dysphagia, restlessness and fright, all of which continued two days. On the fifth day the cicatrized but painless wound became livid, salivary secretion increased and vomiting of bloody material occurred; increased dysphagia and hydrophobia. Respiration, 22. Pulse, 128. Temp., 40° C. Marked excitement, pains in head. Subsequently there supervened delirium, phantasies of death, muscular twitchings, general convulsions, frenzy, and tetanic contraction of the respiratory muscles. Death resulted in one of these tetanic seizures.

Autopsy: The chief findings were congestion and serous imbibition of the pia mater of the brain and medulla, acute internal hydrocephalus, pronounced hyperæmia of the brain cortex and of the grey substance of the spinal cord.

Case 5.‡ Relates to a twenty-six year old man who was bitten in several uncovered places by a rat terrier which had been ill for some days. The dog soon thereafter had convulsions and paretic symptoms in the hind quarters. After its death the mucous membranes of the stomach and intestines were relaxed, intensely reddened and ecchymotic in spots; no normal secretion, but contained much straw, hair, and even small pieces of wood.

* Opus. cit., p. 492.

† Virchow Hirsch Jahresbericht, 1886, Bd. I., 2 te abth. 522.

‡ Virchow Hirsch Jahresbericht, loc. cit.

The man's wounds were on the upper lip, including several small, superficial scratches on the left, and one wound of one and a half cm. in depth on the right hand. They were cauterized with nitrate of silver about fifteen minutes after the production of the injury. Subsequently after being cleaned, were treated with caustic potassa. After suppuration was established the wounds were held clean with a solution of two and a half per cent. of carbolic acid. Healed after seven weeks of suppuration, leaving a red, hard cicatrix. Nine weeks after healing, lyssa developed, with dyspnœa and dysphagia; soon the patient could drink no water; excitement and convulsions supervened, and notwithstanding the patient could be calmed for a time by either rectal injections or the cheerful consolation of the physician, he died after a violent exacerbatian of the hydrophobic symptoms.

Case 6. Rovighi* records a case which presents the peculiarity (?) that the patient, a child, during his hydrophobia bit two persons, who, notwithstanding the fact that their wounds were not cauterized, have not as yet (six months have elapsed) developed the disease. While the clinical history in the child was a perfect one, the autopsy showed no other signs than those of death from asphyxia, though positive results were obtained in a dog who, after being trephined, received a piece of the spinal cord of the child.

These cases are sufficient to show the variable character of the clinical histories, and serve to uphold the statement which we have elsewhere made that no two cases of this alleged infectious disease are alike ; also that the symptom of hydrophobia, considered to be characteristic of the disease by the earlier writers, is not mentioned as such by those of the present day. I may here state that that symptom is considered by the latter to differentiate hysterical lyssa, and lyssophobia from the so-called true lyssa.

Its etiology shows as great discrepancies and absurd statements as could possibly be mentioned. The bite of a dog, wolf, cat, badger, fox, in fact most all the species of the canidæ, mustelidæ and felidæ have been said to produce a disease whose existence originally had only been attributed to the dog, wolf and fox. The animal, some claim, need not be rabid in order to produce the disease ; others deny the same statement ; some assert that the discan originate in the animals named *de novo* ; others, and the most modern scientists, deny this absolutely and ridicule such a belief. It is not necessary, so it is claimed, that there should be any solution of continuity in a tissue to in-

* Sulla transmissibilita della rabbia da uomo a uomo. Revista clinica di Bologna Agosto, No. 8.

troduce the alleged virus, and that the licking of the skin of man by such an animal is sufficient to establish the disease. But we need go no further, for we deny to-day as we denied some years ago, that there is any specific infection from the bite or that the existence of the disease in the dog has been sufficiently well established.

But one suspected case of rabies has ever been seen in the dog pound in New York. It was impossible during the recent scare to obtain a single rabid animal. Indeed, rabid dogs are more rare than cases of lyssa. It is the opinion of Dr. Stockwell* that distemper, toothache, earache, canker, mestoid disease, gastritis, febrile diseases, throat and lung diseases, epilepsy, meningitis, and the whole class of nervous diseases to which dogs are subject are constantly mistaken for it. He says : "Personally, after more than thirty years' experience as physician, dog owner and student of canine and comparative medicine, I have yet to meet with a case of absolute rabies in the dog ; and of some scores of so-called hydrophobic animals presented for my inspection, one and all were found to have suffered from other and comparatively inocuous maladies."

It has been claimed that the disease may arise spontaneously in man.† It is not wonderful that this opinion should have been reached, for so weak is the support of the attributed cause of the disease, that when a case presents itself and it is thereafter shown that the bite was not from a rabid animal or that there was no bite at all, such support must crumble away and leave no other basis than the supposition of an origin *de novo.* If the habit and thought of the medical mind were more analytical, and if physicians would reason and draw conclusions logically, governing their deductions by strict logical laws, many changes would be made in the now accepted opinions of etiology and pathology.

Its morbid anatomy likewise presents no characteristic or even constant feature. From absolutely no changes,

---

* Therapeutic Gazette.

† Girard de Caieleux, Annales Medico Psychologiques, I., 233, 1869.

microscopical or macroscopical, to the most characteristic changes found in exudative meningitis and encephalitis have the findings varied. It is needless for me to repeat the various diseases which have been mistaken for it, as this subject was well nigh exhausted by Dulles of Philadelphia in the discussion on hydrophobia, held before this body a year and one-half ago, and in his instructive paper on the same question.*

Suffice it to say, briefly, that all cases of lyssa may be classified under the following groups: 1. Septicæmia, or blood poisoning. 2. Tetanus. 3. An acute insanity analogous to acute delirium or typhomania. Of course I do not, in this classification, allude to those affections which simulate lyssa, but restrict the above to those cases which have all the symptoms attributed to the usual form of the disease. If lyssa were due to a morbific agent which resides in the secretion of a rabid animal and which is introduced into the circulation of the individual bitten, then the same law of the development of other infectious diseases should govern the development of this disease, that is, a certain definite period of incubation should precede the development of all signs of disease. It is so in scarlet fever, in relapsing fever, in typhoid, in typhus, in small pox and in anthrax. A disease depending upon a period of incubation which varies from a few hours to seventeen years† for its development cannot be due to any infectious element, and the view of specific infection must be regarded as problematical in the extreme.

It is the writer's opinion that if we regard the one form of this disease as a psychosis due to the effects of a strong mental impression, that all the phenomena of this affection can be explained. It is well known that expectant attention can produce physical and psychical phenomena

---

* Disorders Mistaken for Hydrophobia. By Chas. W. Dulles, Philadelphia, 1884.

† Dr. Chas. Bell Taylor, in a recent address before the Nottingham (England) Medical Society, mentions the following: "Two young men, friends, met on the landing stage at Havre, in January, 1853; one was going to America, and both were accidentally bitten by a mad dog. The one that stayed at home died within a few weeks of hydrophobia. The other did not hear of his friend's demise until his return in 1868, fifteen years later; he then took ill himself and died of the same disease."

which have many points in common with lyssa. If we direct our attention to any spot on our surface, we can produce a localized hyperæmia and redness with pain in that locality ; these may be followed by irritability and feverishness, and the changes from being at first local may soon involve the rest of the body.

Nothing is so persistent as a mental impression which has been engendered by a disturbance in the integrity of a tissue ; a healed fracture, a cicatrized wound and an amputated stump, not only from the original impression of the injury but from the continuous attention which such injuries provoke, cannot be eradicted from the memory. What then must the effect of an injury, which has usually been regarded as fatal, be upon a weak mental organization, in whom traditional fear and expectant dread establish a constant memory, displacing and putting in the background the most important psychical functions—functions whose activity is more necessary to the existence of a condition of health than that of any other organ? The more unstable the mental organization of the individual the greater will be the effect of such injuries.

We do not deny that disease and death follow the bite of a rabid dog, but we do deny that the cause of the disease is the nature of a specific poison of invariable qualities introduced into the system.

Our view is that in the great majority of cases a firm, persistent mental impression, combined with expectancy, has so deranged the functional activity of the brain, that some chemical change, perhaps, is formed in the blood which disturbs the equilibrium of the entire nervous axis and results in a set of symptoms which have very much in common with the psychosis which has been variously termed Acute Delirum and Typhomania. I should hence classify certain of the cases reported as lyssa or rabies under the category of the acute insanities and place it with Acute Delirium as a psychosis due to some altered change in the blood. Indeed Acute Delirium is a disease which has also many pathological features in common with lyssa and which has an analogous etiological basis. I have in mind the case of a young girl who came under my care whilst I was house physician at Bellevue Hospital in whom this fatal disease was developed, as a result of the mental impression, which a seduction under promise of marriage and subsequent abandonment by the villain who betrayed her, provoked. There can be no doubt but that this mental impression was the direct cause of the disease which resulted fatally in her case, as it does in as many cases as when lyssa attacks the individual.

Spitzka has taken a similar ground as to the origin of what he calls Grave Delirium and says in a recently published paper* that the diagnosis of this disease is often confounded with that of hydrophobia. He adds further in the same article, "there is a tendency in a number of patients to bite, and this tendency is so strong that in default of a foreign object one such patient bit off his tongue. It is this class of patients who exhibit a terror of water, and undoubtedly many cases of so-called hydrophobia were naught else than grave delirium. To the best of my knowledge it is the only genuine hydrophobia in human pathology."

The case of acute delirum above cited by me showed on the autopsy no other signs than a general hyperæmia of the meninges and engorgement of the cortex, which presented quite a pink color and from which the blood oozed when gently pressed upon. The vessels of the medulla oblongate responded to pressure in a like manner. These are likewise the chief changes, according to reliable observers, which have been noticed in lyssa.

The other similar clinical features are as follows, and what is most suggestive is that these are confined to disturbances in the mental sphere: Irritability of an extreme type, light and sound disturb the patient. He has a fear of impending misfortune and in lyssa often attempts futilely to deny that any danger menaces him. The patient says he is not afraid, he does not fear the bite and its effect (a sign in this condition that he views the injury

---

* Delirum Grave, Journal of the American Medical Association, Aug, 13th, 1887.

with dread, or at least that the impression on his mind is fixed). Both frequently break forth into acts of violence, usually more aggressive in the hydrophobic than in the one suffering from acute delirium. These aggressive acts manifest themselves by snapping at surrounding objects, biting whatever may be near them, using their fists. There is an expression of countenance denoting dread and fear. Delirium is a more marked feature in the latter than in the patient with hydrophobia, although there it exists very frequently. Physical prostration is extreme in both. Hallucinations and illusions are sometimes present; and in both there are quiet intervals in which the reason may relatively clear. Insomnia is present in both. These signs are sufficient to stamp lyssa as a species of acute delirium or as an independent affection analogous to it.

The period of incubation is as variable in the one as in the other, that is if we may speak of such a period in reference to a disease which we cannot, in the light of this view, consider as infectious.

It is only those of a relatively weak mental organization who succumb to this disease, for its etiological factors have a greater influence on such persons. Indeed the mortality from lyssa is less than three in every million of the inhabitants in those countries where it is most prevalent. This percentage becomes greatly increased as soon as those factors, which tend to bring the disease before the public mind, and to pander to morbid sensationalism, operate. This is a medico-legal inquiry which should be well considered and in our opinion on which some action should be taken. The following question might arise in reply to our statement that expectant attention, and the emotions born of the instinct for self-preservation were the most important factors in the development of this mental affection: How can the reported cases of lyssa in children and infants be accounted for? The answer to this is very readily furnished by a perusal of the histories of reported cases. It will be invariably found that either the disease has affected a child who was old enough to have learned to fear; whose mind was excited by the stories of mad dogs, bites in others, and kindred topics; or being too young to be influenced by any mental impression, the condition called erroneously hydrophobia will on analysis prove to be one of tetanus* or septicæmia.

If the press was aware that it is the chief cause of so called epidemics of rabies, and that by eliminating from its columns all accounts of cases of this disease, it could control and diminish its prevalence, the mortality from lyssa would be almost *nil*. Much good could be done by the same medium in instructing the people that there is no more danger from the bite of a dog than from that of a man ; that there is no specific infection in such a bite, and that most cases of lyssa are but the expression of a morbid mental state, in other words are an insanity, which affects those individuals in whom fear, dread, or expectant attention have obtained the ascendancy over those other mental faculties, whose activity is most necessary to a condition of mental integrity. It has been the history of this disease that whenever public attention has been called to its terrors that the number of cases increased with a remarkable regularity.

The premature sensational publicity given to Pasteur's method is therefore likewise responsible for many deaths, not only directly but indirectly. Directly, as numerous cases attest, by the introduction of a poison into the system ; indirectly by bringing to public attention an alleged discovery of a method of treatment whose efficacy is now established to be extremely doubtful.

Germany has treated his announcement, as it treats everything that is new, with cautious reserve. A few have viewed his methods with scepticism, and concluded that his *pronunciamentos* should be treated with indifference. One cannot blame them for arriving at this conclusion, for in addition to their discovery of the unrelia-

* One of these cases diagnosed as hydrophobia, and wrongfully so, is reported by Dr. A. H. Foster, Journal of Nervous and Mental Diseases, 1874, and in the Annales de Medico-Psychologiques, Vol. I., p. 464, 1879. The patient was an infant who had been bitten by a supposed rabid dog. Death resulted in 60 hours being preceeded by convulsions induced by the presentation of food or drink, and by a current of air, and the walking of a fly over the face. There were no mental symptoms. This could have been nothing else than a case of tetanus.

bility of the so-called French Jenner they have had an experience with the results of another alleged discovery of the same individual, viz.: his preventive inoculation against anthrax in cattle. That the former subject has not received more than an indifferent recognition by the majority of German scientists may be explained when we consider that careful investigators had carried out his process of vaccinnation for anthrax, and with material which he himself furnished, and found just as many deaths from this disease in the vaccinated as occurred in the unvaccinated cattle.

The commission appointed by the Belgian Government to examine and report upon his method gave an unfavorable decision. The request for the establishment came from the Chamber of Deputies. The Commission was formed of three Belgian physicians residing in Paris—Drs. Grandjean, De Bruyn and Peters. It was decided by them that "Pasteur's method is not as yet sufficiently established, and one of them, Dr. De Bruyn, doubted whether there was any efficacy at all in the treatment. In this last view Van der Corput, Belgian's chief specialist, coincides."*

The Austrian Government has withdrawn its support. The Pasteur Hospital at Vienna is begging for funds, and having applied (Journal de Médicine de Paris) to the Austrian Minister of the Interior for assistance, was refused with the statement that the numerous failures of the alleged preventative inoculation against rabies had induced him, after a careful investigation, to deny the grant of any aid to the hospital. Likewise the directors of the "Austrian Hospital have decided to suppress preventative inoculation in future, as they fail to give any good result."†

In fact there was, not long ago, a tendency in the Paris municipal government to question the efficacy of Pasteur's method; for the Paris Municipal Council have under consideration a resolution offered by M. Chaissang as follows: "In consideration of the fact that M. Pasteur has altered

* Med. and Surg. Reporter, July 9, 1887.

† Chicago Medical Standard, Oct., 1887, p. 106.

his treatment and substituted his so-called intensified method, and since the introduction of this method not a week passed without cases of madness known as the '*Rage de Laboratoire*' having declared themselves, and as it is evident that M. Pasteur's system, instead of producing favorable results, is a real danger, it is now proposed that the Council do revoke its previous votes in favor of the Pasteur Institute, and M. le Prefect is invited to take steps to suspend their execution."*

Had Pasteur had an honest faith in the reliability of his own work, he would never have deviated from the position which he first took when he promulgated his alleged discovery to the world. But the mere fact that he has so frequently changed his statements, first in reference to his assertion that he could protect all and every case of the bitten that would present themselves to him, next, in reference to the so-called period of incubation, and, finally, to the very method of treatment itself, ought to produce a doubt as to the correctness of his observations and results. When cases that were inoculated by him presented symptoms of the disease after treatment, he asserted that the inoculated material was not of sufficient strength to destroy the effect of the bite. He thereupon used a stronger virus (intensified method) and soon discovered that he was introducing into the system of the patients under his care a virus which destroyed life; he therefore again changed his position and asserted that patients required a virus of an intermediate strength to combat the original effect of the infection, and at present has rested his claims upon this method, stating that his intensified method was without doubt protective.

But the most inexplicable feature of this is the confidence with which he maintains each of his changed positions. This is more characteristic of the methods of a pure empiric or of a charlatan than that of a scientific observer who desires to have nothing but the truth to prevail.

---

* Pasteur's Prophylactic, an address delivered at the meeting of the Nottingham Medico-Chirurgical Society, March 18, 1887. By Charles Bell Taylor, M. D., F. R. C. S.

Pasteur's chief support is now in Russia, in Brazil, in England and, I believe, in Siam. It is not necessary to analyze the cause of this support in any of these countries —countries where no trustworthy scientific work is done, except in the case of England.

An analysis of the report of the British Hydrophobia Commission will disclose the following facts, which can only be regarded as opposing its own conclusions; and it will also be found that none of the questions at issue has been satisfactorily and properly examined.

In the first place, there is as much doubt about who of this committee went to Paris to examine Pasteur's methods, since the British *Medical Journal*, July 2d, 1887, and the London *Lancet* of the same date, have different statements on this subject,* as there is in regard to the author of the report. There is great *prima facie* evidence that this was chiefly the work of Mr. Victor Horsley, the Secretary of the Commission, and the one who performed the experiments by which the Commission came to its conclusions. The fact that this report, based upon the work of one man, and that man one who has ever been a pronounced partisan, was be signed by the other members, giving the impression that it was the result of the entire Commission's labors, should be sufficient to stamp it as unworthy. Had the work been done by the various commissioners and their results as individually interpreted been correlated, we might place some credence upon their conclusions. As it is the report has numerous deductions which could not be reached from the premises, showing not only fallacious argumentation, but almost a sophistical tendency. But let us examine the report as it is.

One of the conclusions of the report is based upon the following experiment:

Two rabbits had been inoculated by Pasteur during the visit of the Committee, and were brought back to England, where they were kept at the institution of which Mr. Horsley is superintendent. Here, within a week, they developed the symptoms which have been ascribed to hydrophobia in the rabbit. The chief of these is alleged to be "ascending paralysis."

---

* The former asserts that Prof. Burdon Sanderson, Dr. Lander Brunton and Mr. Victor Horsley were the only members that went to Paris for this purpose, the latter that Sir Henry Roscoe formed one of the party of three.

The symptoms of hydrophobia in the rabbit are vague and indefinite. No two authors and experimenters have found them to be the same. We have been able to produce paralysis in a rabbit by inoculation with healthy spinal cord which had been treated in the manner described by Pasteur. Indeed Pasteur himself has said that the rabbit is not a test animal to experiment with.* Still he makes use of them. However, we will accept that these animals of Mr. Horsley had the signs of hydrophobia.

The spinal cords of these Paris rabbits were inoculated beneath the dura mater of four other rabbits at the Brown Institution and they developed the same symptoms in about seven days; four dogs were inoculated with the same material and developed rabies, according to the report, in eight days. In two of the latter the disease assumed the form of furious, and in the remaining two, that of dumb rabies.

In a series of experiments made a little over a year ago, in connection with Dr. E. C. Spitzka, we found that we could produce the symptom-complex assigned to hydrophobia in rabbits by inoculating with septic material, or with the healthy spinal material of another rabbit; but the symptoms varied, with the exception of paralysis, which was constant in each, almost as often as the number of experiments. Wishing then to have the followers of Pasteur accurately define their position as to what was true rabies in the rabbit, the question was asked by Dr. Spitzka of Mr. Victor Horsley, who was the most outspoken of Pasteur's supporters, what are the symptoms in the rabbit which characterize the disease.† Need we say that no answer was given in the reply except to refer the questioner to Pasteur. Well, such is the fact, and it is not to be wondered at, for we can assure you that all the symptoms which have been alleged to constitute rabbit rabies, we can produce in rabbits at will. To continue: we utilized the spinal cords of these paralytic rabbits prepared in strict accordance with Pasteur's directions and inoculated them into dogs, and produced in the dogs those symptoms which have been ascribed to dumb rabies in the dog. In other

*Dr. H. W. Biggs of the Carnegie Laboratory, who witnessed Pasteur's experiments, stated decisively before this society that the rabbit has no characteristic signs of rabies.

†Letter in British Medical Journal, 1886.

dogs healthy spinal cord of the calf was used with the same results. Then indifferent substances like so .p, saliva, etc., were introduced under the dura mater cerebri and like results attended the experiments. The detailed accounts of a few of these were presented to you in June, 1886,* when this subject was exhaustively treated by the doctor. In the light of these results we cannot accept the conclusion of the Parliamentary Commission as justifiable or correct.

The same experimenter of the British Commission is said to have then exposed some rabbits to the bites of dogs found in the streets suffering from furious or dumb rabies, and produced paralytic rabies in the rabbits, and inoculation with the spinal cords of these dogs produced the same effect. It is very curious, that dogs suffering from rabies, which is very rare according to the opinions of some of the most expert English veterinarians, should have most conveniently presented themselves to Mr. Horsley at the precise moment when he most needed them to sustain the position which he takes.

These members of this Commission investigated in Paris the history of ninety patients. They could find no reliable evidence of the fact in thirty-one cases, that the dogs who bit them were rabid; in others the bites were inflicted through the clothes, but in twenty-four cases bites had injured exposed parts, and were made by "undoubtedly rabid dogs, and the wounds were not cauterized or treated in any way likely to have prevented the action of the virus."* None of these ninety have yet died of hydrophobia, but the Committee report that they believe that eight would have died if the inoculations had not taken place. By what process of reasoning do they come to this conclusion? It is very unlikely that a person having been bitten by what he supposes to be a rabid animal would delay treatment of some sort until he could reach the presence of M. Pasteur. Sucking of the wound, cauterization or some such means would be undoubtedly the first thought to be

* Journal of Comparative Medicine and Surgery, July 1886, pp. 261, *et seq.*

† British Medical Journal, loc. cit.

put into effect; yet the report says that nothing was done until they came into M. Pasteur's hands. We can throw out of consideration the thirty-one who were bitten by non-rabid dogs and the others who were bitten through their clothes, as no infection (?) could have entered the wounds, so that the Committee are responsible for the statement that eight would have died out of the twenty-four who were infected.

Youatt employed cauterization in over 400 persons bitten by rabid animals and there was not a single patient of these that died from lyssa.

A surgeon at St. George's Hospital treated 4,000 cases of persons who had been bitten by presumably rabid animals and declared before a Committee of the House of Commons in 1830 that he had cauterized the wounds without a single case of hydrophobia developing.*

Of 233 persons bitten by rabid dogs in Zürich, during a period of forty-two years, only four developed the disease and died. Of 106 cases who presented themselves for treatment at the Hospital in Stockholm during the epidemic there in 1824, only one case showed any signs of disease.† Of Hunter's twenty-one cases only one developed the disease, likewise but one of the twenty reported by Vaughn.

Admitting then even the number indefinitely spoken of in the report as "in others" as having been bitten through their clothes, which ought to have served to rub off the saliva from the teeth had it carried any infection, the report claims that Pasteur's treatment saved at least eight lives out of fifty-nine people that have been bitten. On what basis do they calculate? What statistics do they use to determine the number who would have developed the disease from the bite? The last question is as unsettled as all the other features of the disease. The most authors, and the report itself, assert that five per cent. of persons bitten by rabid dogs develop the disease. Making a gen-

* Reynold's System of Medicine, Vol. I., p. 335; Macmillan & Co., London, 1876.

† Zur Medicinischen Statistik, J. Appella, p. 9, 1886. Berlin, H. S. Hermann, publisher.

erous allowance then we will say for the sake of argument that ten per cent. of the fifty-nine people whose histories were examined might have developed lyssa, in other words, five would have died from the disease. Yet the Committee are assured that eight would have died had inoculations not been practised.

The report also says that of 233 persons bitten by animals in which rabies was proved only four died ; without inoculation 40 would have died.

Comparing this statement with the cases occuring at Zürich, previously mentioned, we find that by a strange coincidence the number of cases is the same as is the number of deaths: 233 persons bitten and yet four deaths in each total. If we accept, therefore, the deductions of the Commission, thirty-six people in Zürich who were bitten had no right to live. We would, therefore, kindly request these survivors, if they have not as yet "shuffled off this mortal coil" from other causes, to cheerfully assist the British Hydrophobia Commission's cause and hastily take some means to depart from earth's pleasures ; since having been bitten by rabid dogs and not having been inoculated according to the most approved methods, they live yet have no license to live.

The report continues to say : " making fair allowance for uncertainties and for *questions that cannot now be settled*, * we believe it sure that excluding the deaths after bites from rabid wolves, the proportion of deaths in the 2,634 persons bitten by other animals was between 1 and 1.2 per cent., a proportion far lower than the lowest ever estimated among those not submitted to M. Pasteur's treatment, and showing even in its lowest estimate the saving of not less than one hundred lives."

If there were no deaths in the 400 cases of Youatt whose wounds were only cauterized, and 31 deaths in Pasteur's 2,634 who were preventively inoculated, in whose favor is the percentage and where was there "a saving of not less than one hundred lives?" Where the Committee in the other portion of their report make mathematical calculations they show themselves just so deficient in arithmetical knowledge or ignorant of statistics, for they make calculations in a similar infelicitous manner.

---

* Italics mine. What may these questions be? What a scientific and accurate method of analytical deduction !

The worst feature of the report, and the one which would alone militate against its conclusions, is the attempt to explain the death of an attendant of the Brown institution, Goffi by name, who was bitten on Sept. 4th, 1886, and was under M. Pasteur's care in Paris the very next day. He died at St. Thomas' Hospital on Oct. 19, 1886, from acute ascending (motor) paralysis which Horsley said is a form of hydrophobia. That the symptoms of lyssa should be so narrowed down and restricted that a case of Landry's paralysis should be called hydrophobia, so that the position of the advocates of Pasteur might be sustained, needs no further comment The Commission, through Mr. Horsley, determined then that Goffi died of Landry's paralysis, which was an example of the paralytic form of rabies in man ; that he hence died as a result of the bite of the cat, who was the infectious animal in this instance. If that be so, then the intensive inoculations which had been used in this case, and which were considered by the Commission to be efficacious, must be worthless, and the report thus contradicts itself.

It requires no analytical mind to perceive from this that instead of the report sustaining Pasteur, which is its acknowledged intention, it furnishes the mightiest and most important elements to destroy his position. For if the intensive method be thus demonstrated to be unreliable, what trust shall we put on the intermediate, the present method of treatment.

The only good feature in the report is its suggestion as to police regulations. Its recommendations are :

1. Destruction of all wandering dogs.
2. High taxation which would thus discourage the keeping of them.
3. Prohibitive importation from countries where rabies prevails (?)
4. Compulsory use of muzzles.

With the exception of the third of these recommendations, I should likewise urge their adoption in this State, and it is to be hoped that the Legislature will be impressed with the importance of the results achieved thereby. For the public mind is not yet educated to accept our belief as to the non-specific character of the dog bite, but which for its own sake I trust will soon occur. The press ought to be the medium of instruction for this conversion. Bavaria has instituted a most stringent dog law. It compels owners of dogs, under threat of immediate death to the animals, to affix upon the collar of the dogs a metal device upon which is inscribed legibly the number of the dog as registered in his owner's district. The color and shape of this metal inscription is changed every year, so that at a glance it may be seen whether the dog is all right as to its registration. In addition, examinations of dogs must be made monthly and by competent veterinarians, and if there is the slightest sign of any illness whatever, the animals are sent to a canine hospital and detained there until they recover. If the ownership of an animal should be changed the police are at once notified. Any breach in any of these regulations—even a few days' delay in paying the tax—is punished severely. As a result of these measures out of a population of 6,000,000 there have been but three deaths in the last seven years ascribed to lyssa.*

Should these laws be adopted here, and should the press take upon itself the position of teacher to the multitude and instruct them in the manner that I suggested, and unite in the determination to omit from their columns all sensational accounts of the disease, the writer is sure that hydrophobia, like its ancestor, lycanthropy, will be relegated to the domain of the past and no longer furnish an element in the necrology of the human race, but present itself only as an atavism at some future day in the mental disorders of those descendants who may be unfortunately smitten with insanity, just in the same manner that we now meet, rarely, it is true, a case of insanity having lycanthropic delusions.

* The Medical Register, Philadelphia, Sept. 24, 1887.

